# Negligible contribution of adaptation of ocular opponency neurons to the effect of short-term monocular deprivation

**DOI:** 10.3389/fpsyg.2023.1282113

**Published:** 2024-01-11

**Authors:** Jue Wang, Fangxing Song, Xin He, Min Bao

**Affiliations:** ^1^CAS Key Laboratory of Behavioral Science, Institute of Psychology, Chinese Academy of Sciences, Beijing, China; ^2^Department of Psychology, University of Chinese Academy of Sciences, Beijing, China; ^3^State Key Laboratory of Brain and Cognitive Science, Beijing, China

**Keywords:** short-term monocular deprivation, ocular dominance, opponency neuron, plasticity, steady-state visually evoked potential

## Abstract

**Introduction:**

Modeling work on binocular rivalry has described how ocular opponency neurons represent interocular conflict. These neurons have recently been considered to mediate an ocular dominance shift to the eye that has viewed a backward movie for long during which time the other eye is presented with a regular movie. Unlike typical short-term monocular deprivation, the visual inputs are comparable across eyes in that “dichoptic-backward-movie” paradigm. Therefore, it remains unclear whether the ocular opponency neurons are also responsible for the short-term monocular deprivation effect which is prevalently explained by the homeostatic compensation theory. We designed two experiments from distinct perspectives to investigate this question.

**Methods:**

In Experiment 1, we mitigated the imbalance in the activity of opponency neurons between the two eyes during monocular deprivation by presenting video stimuli alternately. In Experiment 2, we directly evaluated the response of opponency neurons before and after monocular deprivation using SSVEP techniques.

**Results:**

Consistent with each other, both experiments failed to provide reliable evidence supporting the involvement of ocular opponency neurons in the short-term monocular deprivation effect.

**Discussion:**

Our results suggest that ocular opponency neurons may not play an essential role in the short-term monocular deprivation effect, potentially due to interference from the homeostatic plasticity mechanism.

## 1 Introduction

Ocular dominance refers to the functional asymmetry between the two eyes, which was believed to be established during the critical period of development and hardwired in adulthood (Wiesel and Hubel, 1963). However, previous research has revealed that the visual system of adults retains a degree of ocular dominance plasticity by showing that a few hours of visual deprivation on one eye will induce a shift of ocular dominance to the deprived eye (Lunghi et al., 2011). Since then, short-term monocular deprivation and its underlying mechanisms have been extensively investigated (Lunghi et al., 2015; Bai et al., 2017; Kim et al., 2017; Min et al., 2019; Lyu et al., 2020; Nguyen et al., 2021).

One suggested mechanism for monocular deprivation is homeostatic plasticity (Baroncelli and Lunghi, 2021; Bang et al., 2023), an inherent mechanism that maintains the normal functioning of the neural system by stabilizing neuronal activity (Keck et al., 2017). To accomplish this, the mechanism resets the neural system to its baseline state following a perturbation, preventing the system from becoming hyperactive or hypoactive (Turrigiano, 1999; Turrigiano, 2011). Homeostatic plasticity in the visual system was initially observed in monocular deprivation studies in rodents (Maffei et al., 2004; Turrigiano and Nelson, 2004). In the context of short-term ocular dominance plasticity, an imbalance in visual input between the two eyes may trigger a homeostatic upregulation of neural response in the deprived eye to maintain the balance of neural activity within the visual system. This can lead to a shift in ocular dominance towards the deprived eye following the monocular deprivation (Lunghi et al., 2015; Bai et al., 2017; Binda et al., 2018; Min et al., 2019; Zhou et al., 2019; Lyu et al., 2020).

Besides monocular deprivation, recent work has developed new methods to investigate short-term ocular dominance plasticity (Spiegel et al., 2013; Wang et al., 2021; Huang et al., 2022; Tuna et al., 2022; Song et al., 2023b). Song and colleagues invented a “dichoptic-backward-movie” adaptation paradigm to study the ocular dominance shift induced by sustained eye-based attention. The paradigm involved presentation of a movie played normally to participant’s one eye (attended eye) along with the same movie played backwards to the other eye (unattended eye). Adaptation to such stimuli heightened the unattended eye’s dominance in the binocular rivalry task, reflecting a shift of perceptual ocular dominance towards the unattended eye. Moreover, they minimized the interocular conflict during the adaptation to further investigate the role of eye-based attention and observed no shift in ocular dominance (Song et al., 2023b). This finding implies that the attention-induced ocular dominance shift may not be explicable solely by the homeostatic plasticity mechanism, because the involvement of homeostasis is not dependent on interocular competition (Zhou et al., 2013; Ramamurthy and Blaser, 2018; Min et al., 2022).

**Figure 1:**
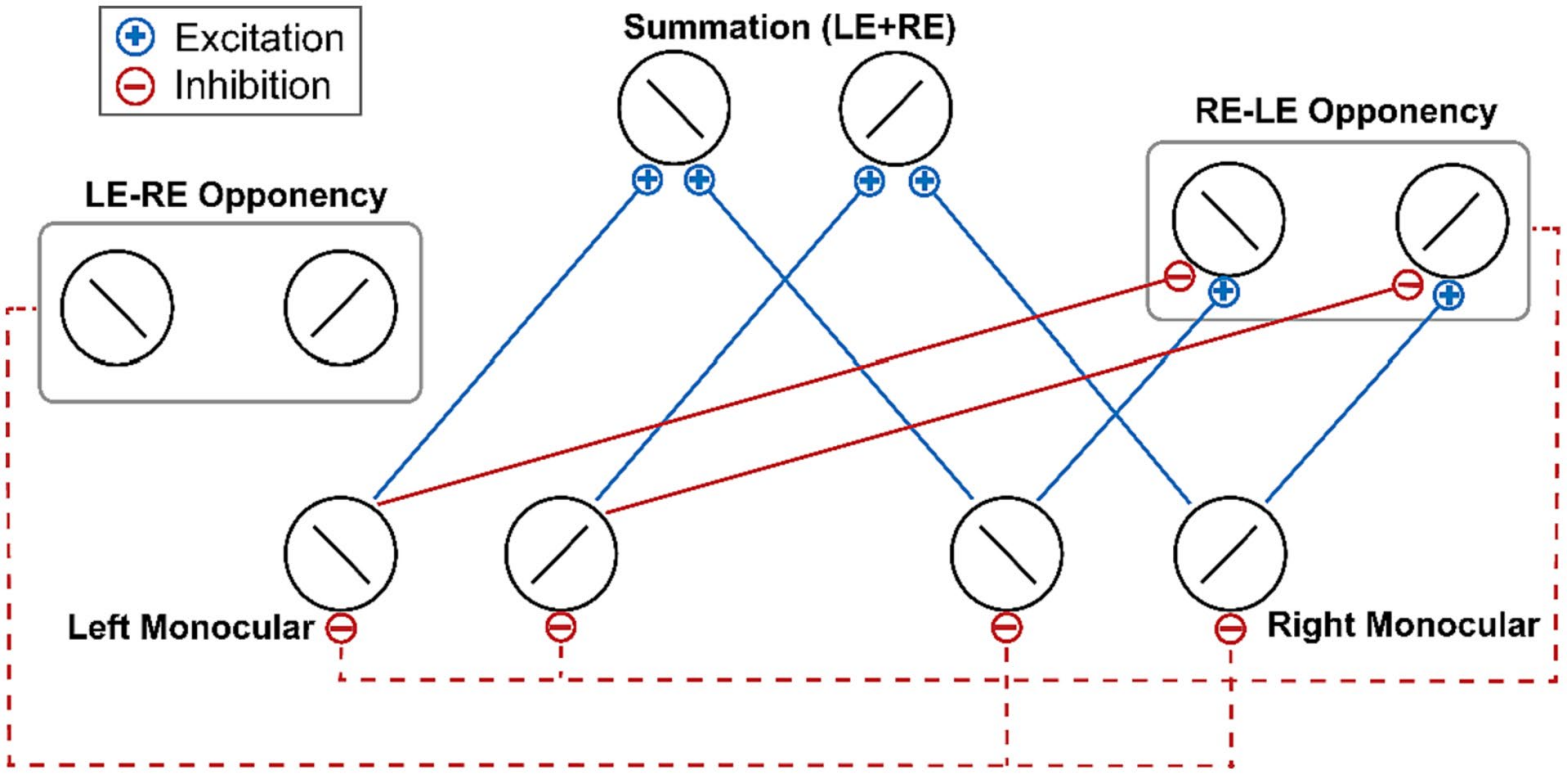
The schematic of the ocular-opponency-neuron model (Said and Heeger, 2013). Solid blue lines denote excitatory monocular input signals. Solid red lines denote inhibitory monocular input signals. Dash red lines denote the suppression of the opponency neurons to the activity of monocular neurons. For brevity, the figure does not depict the excitation and inhibition inputs received by the LE-RE opponency neuron. LE, left eye; RE, right eye.

Given the crucial role of interocular competition in the effect of attention-induced ocular dominance shift, Song et al. (2023a) proposed an explanation based on the ocular-opponency-neuron model (Said and Heeger, 2013) of binocular rivalry. According to the ocular-opponency-neuron model, each opponency neuron receives excitatory inputs from a monocular neuron for one eye and inhibitory inputs from a monocular neuron for the other eye. Then a difference signal is computed and half wave rectified so that the opponency neuron fires only when the excitatory inputs surpass the inhibitory inputs. Upon activation, the opponency neuron, in turn, inhibits the monocular neuron that sends the inhibitory signals to it (Figure 1). Song and colleagues hypothesized that during the adaptation phase, the ocular opponency neurons may undergo different degrees of adaptation between the two eyes, ultimately resulting in imbalanced inhibition and thus a shift of ocular dominance (Song et al., 2023b).

It should be noted that the content of visual input is comparable across eyes in the dichoptic-backward-movie paradigm, yet the input in one eye is completely or partially lost in a typical short-term monocular deprivation paradigm. Considering that the two paradigms are obviously different from each other, it remains unclear whether the ocular opponency neurons are also responsible for the effects of typical short-term monocular deprivation which was prevalently explained by the homeostatic compensation theory (Lunghi et al., 2011; Zhou et al., 2013; Bai et al., 2017; Kim et al., 2017; Lyu et al., 2020; Wang et al., 2020; Nguyen et al., 2021). Therefore, the purpose of this study is to examine the contribution of ocular opponency neurons in the shift of ocular dominance induced by short-term monocular deprivation. The monocular deprivation in Experiment 1 was based on the paradigm employed in our prior studies (Bai et al., 2017; Lyu et al., 2020; Song et al., 2023b). We measured the changes in ocular dominance before and after monocular deprivation utilizing two different forms of stimuli. Specifically, we modified the traditional continuous monocular deprivation to rebuild a segmented monocular deprivation approach, which involved segmenting the adaptation video into short intervals separated by black screens. During adaptation, the original video images and phase-scrambled images were presented to the non-deprived eye and deprived eye, respectively, either simultaneously (“Simult” condition) or alternately (“Altern” condition). In the “Simult” condition, the phase-scrambled image and the original image were presented at the same time followed by a black screen to both eyes, which repeated for a total time of 1 h. And in the “Altern” condition, the black screen alternated between the two eyes to make sure that only one eye was receiving the images at any time.

Different observations can be anticipated based on the two different hypotheses mentioned above. From the perspective of the homeostatic compensation theory, both eyes were exposed to stimuli for the same amount of accumulated time throughout the monocular deprivation phase regardless of presentation conditions, and the neural activity was stronger in the eye that viewed normal images compared to the eye that viewed phase-scrambled images. Hence, we expected to observe a shift of ocular dominance in both conditions without a significant difference in between. In contrast, according to the opponency-neuron-adaptation hypothesis, the deprivation effect will be attenuated or eliminated in the “Altern” condition (compared with the “Simult” condition) because the degrees of adaptation of ocular opponency neurons were comparable between the two eyes in this condition. Specifically, the alternating presentation of video images between the two eyes in the “Altern” condition yielded comparable periods of dominance for each eye. Consequently, the ocular opponency neurons for each eye activated for similar durations, thereby resulting in a more balanced degree of adaptation among these neurons compared to the “Simult” condition.

It should also be noted that although the design of the current Experiment 1 bears resemblance to that of Song et al.’s (2023a), they fundamentally differ from each other. The present study employed a monocular deprivation paradigm to investigate the role of ocular opponency neurons in the effects of typical short-term monocular deprivation, while Song et al. (2023a) utilized a “dichoptic-backward-movie” adaptation paradigm to examine the contribution of ocular opponency neurons in attention-induced ocular dominance shift without unbalancing the strength of visual inputs between eyes. These two paradigms have proved to yield different effects in terms of neural ocular dominance (Lunghi et al., 2015; Lyu et al., 2020; Song et al., 2023a).

## 2 Experiment 1

### 2.1 Materials and methods

#### 2.1.1 Participants

Fifteen adult participants (13 females, age range 18–28 years) participated in experiment 1. All participants were naive to the experimental hypotheses and had normal or corrected-to-normal vision (actually measured via visual acuity test). The numbers of participants were predetermined based on the sample sizes of published studies in this field (Lunghi et al., 2013; Yao et al., 2017; Min et al., 2018; Sheynin et al., 2019).

#### 2.1.2 Apparatus

The experimental stimuli were programmed in MATLAB and Psychtoolbox (Brainard, 1997; Pelli, 1997) and displayed on a gamma-corrected 27.2-inch ASUS VG278HE LED monitor (refresh rate: 120 Hz, 1920 × 1,080 pixels) with a mean luminance of 76.19 cd/m^2^. We also used the NVIDIA 3D Vision Pro system to present dichoptic stimuli. All participants were instructed to wear the 3D Vision Pro Glasses to view the screen, ensuring that the visual stimuli presented to the two eyes were different. The visual distance from the monitor to participants’ eyes was 70 cm. A chinrest was used to help minimize head movement and the experiments were conducted in a dark room.

#### 2.1.3 Stimuli and procedure

##### 2.1.3.1 Binocular rivalry test

The stimuli used in the experiment consisted of two sinusoidal gratings (Michaelson contrast: 80%, diameter: 1°, spatial frequency: 3 cpd). The orientation of gratings was 45° either clockwise or counterclockwise. These stimuli were presented dichoptically, precisely at the center of the visual field for each eye. Additionally, to facilitate stable binocular fusion, a central red fixation point with a diameter of 0.07° and a high-contrast checkerboard frame (size: 2.5° × 2.5°, thickness: 0.15°) were presented to both eyes.

Each binocular rivalry test consisted of sixteen 60-s rivalry trials. Within each trial, a 5-s blank interval was presented initially, followed by the presentation of rival gratings for a duration of 55 s. The orientation and contrast associated to each eye remained constant throughout a single trial but were randomized across different trials. Participants were given instructions to hold down one of the three keys (Right, Left, or Down Arrow) to indicate their perceptions, which corresponded to clockwise, counterclockwise, or mixed perceptions, respectively.

##### 2.1.3.2 Monocular deprivation

Monocular deprivation was achieved by removing the Fourier phase regularity of input images in one eye (Bai et al., 2017; Lyu et al., 2020). In this paradigm, the achromatic video images were always presented to one eye (i.e., the non-deprived eye), whereas the phase-scrambled images of each video frame were presented to the other eye (i.e., the deprived eye). The phase-scrambled image was generated by replacing the Fourier phase spectrum of the original video image with the phase spectrum of a white noise image (randomly selected from 30 pre-defined white noises every 2–5 s). We referred to the phase-scrambled images as “pink noise.” The power spectra of pink noise images were exactly the same as those of the video images and their difference was less than 10^−5^%. During the adaptation phase, the stimuli were surrounded by a checkerboard frame (size: 39.40° × 24.06°; thickness: 0.08°). The frame rate of the video was 30 Hz.

**Figure 2:**
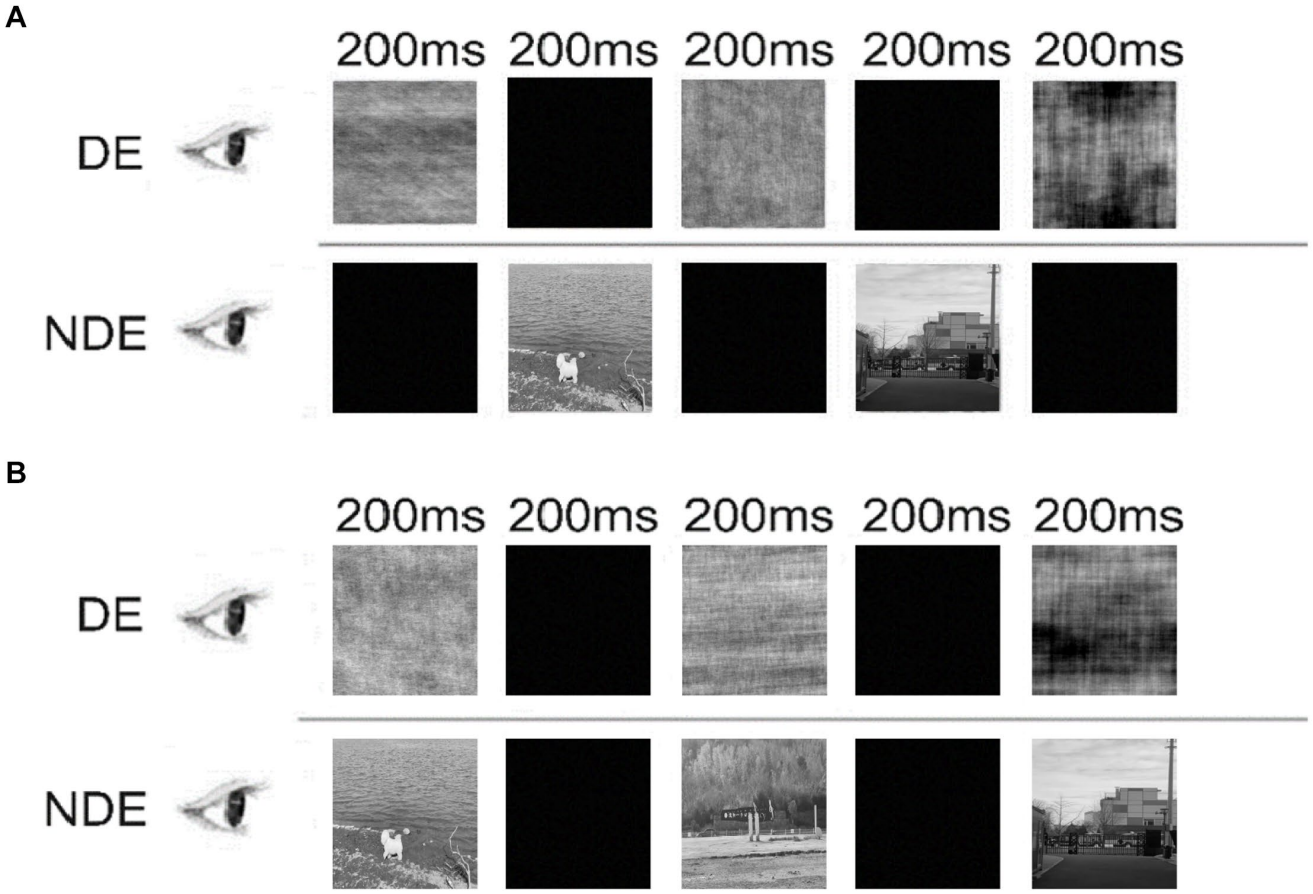
Illustration of the “Altern” condition **(A)** and “Simult” condition **(B)** in Experiment 1. DE, deprived eye; NDE, non-deprived eye.

We designed two different monocular deprivation conditions (Figure 2). In the “Altern” condition, the achromatic video images and pink noise images were presented to the non-deprived eye and deprived eye, respectively. Importantly, their presentations were alternated with each other every 200-ms (Figure 2A). Thus, at any time, only one eye was stimulated while the other eye viewed a black screen. By contrast, in the “Simult” condition, the video images and pink noise images were simultaneously presented to the two eyes, respectively, for 200 ms followed by a 200-ms black screens to both eyes, and so forth (Figure 2B).

#### 2.1.4 Experimental design

Prior to the formal experiment, all participants underwent a training period for the binocular rivalry task lasting from 3 to 7 days to ensure stable performance (Bao et al., 2018). During this training period, participants were required to complete four binocular rivalry tests each day, with a 10-min intermission between tests (except for a 5-min break following the first two tests). The initial test, comprised of five trials, served as a warm-up and was not included in the subsequent data analysis (Bai et al., 2017; Bao et al., 2018). The remaining three tests each contained 16 trials. The determination of perceptual eye dominance was based on the results from the three binocular rivalry tests conducted on the final day of practice, with the dominant eye identified as the one exhibiting longer summed phase durations.

In the formal experiment, the participant had to complete a pre-test of binocular rivalry, followed by 1 h of monocular deprivation, and finally a post-test of binocular rivalry. During the monocular deprivation, the dominant eye was always deprived. That is, pink noise images were always presented only to the dominant eye. Because each condition had to be repeated for three times, participants completed the formal experiment on six separate days. Eight of the 15 participants completed the “Simult” condition for three times firstly, followed by the “Altern” condition. The remaining seven participants did the opposite.

#### 2.1.5 Data analysis

To quantify perceptual eye dominance, we calculated the ocular dominance index (ODI) for the pre- and post-test of binocular rivalry. The ODI was calculated using the following formula, with scores ranging from 0 (complete dominance of the non-deprived eye) to 1 (complete dominance of the deprived eye). $T_{DE}$ and $T_{NDE}$ represented the summed durations for exclusive perception of the stimulus in the deprived and non-deprived eye, respectively.


$$ODI=\frac{T_{DE}}{T_{NDE}+T_{DE}}$$

Statistical analyses were performed using MATLAB. To investigate the effects of short-term monocular deprivation on ocular dominance, the ODIs of the “Simult” condition and of the “Altern” condition was compared between pre- and post-tests using a 2 (condition: “Simult” and “Altern”) × 2 (test phase: pre-test and post-test) repeated measures ANOVA. Post-hoc tests were conducted using paired *t*-tests, and the resulting *p*-values were corrected for multiple comparisons using the false discovery rate (FDR) method. All *t*-tests were two-tailed and an α value of 0.05 was used.

Furthermore, we complemented the standard inferential approach with the Bayes factor (Wagenmakers et al., 2018; van Doorn et al., 2021; van den Bergh et al., 2023), which allows quantifying the relative evidence that the data provide for the alternative (H_1_) or null hypothesis (H_0_). The Bayesian analyses were conducted using JASP with default priors. For Bayesian paired *t*-tests, we computed *BF*_10_ which suggests the probability of the data to be generated under the H_1_ compared to the H_0_. For Bayesian repeated measures ANOVA, we computed inclusion Bayes factors (*BF*_incl_) which suggest the evidence for the inclusion of a particular effect calculated across matched models. A BF greater than 1 provides supports for the alternative hypothesis. Specifically, a BF indicates weak evidence if between 1 and 3, moderate evidence if between 3 and 10, or strong evidence if greater than 10 (van Doorn et al., 2021). Similarly, a BF below 1 provides evidence in favor of the null hypothesis.

### 2.2 Results

Perceptual ocular dominance was measured before and after monocular deprivation. The repeated measures ANOVA revealed a significant main effect of test phase (*F*(1,14) = 14.36, *p* = 0.002, *η*^2^ = 0.51). The Bayesian ANOVA also indicated moderate evidence for the test phase effect (*BF*_incl_ = 4.782). However, neither the main effect of condition (*F*(1,14) = 0.80, *p* = 0.386, *η*^2^ = 0.05, *BF*_incl_ = 0.691) nor the interaction of condition × test phase (*F*(1,14) = 0.99, *p* = 0.338, *η*^2^ = 0.07) was significant. The Bayesian ANOVA yields weak evidence (*BF*_incl_ = 0.525) supporting the null hypothesis of no interaction effect between the test phase and the condition.

**Figure 3:**
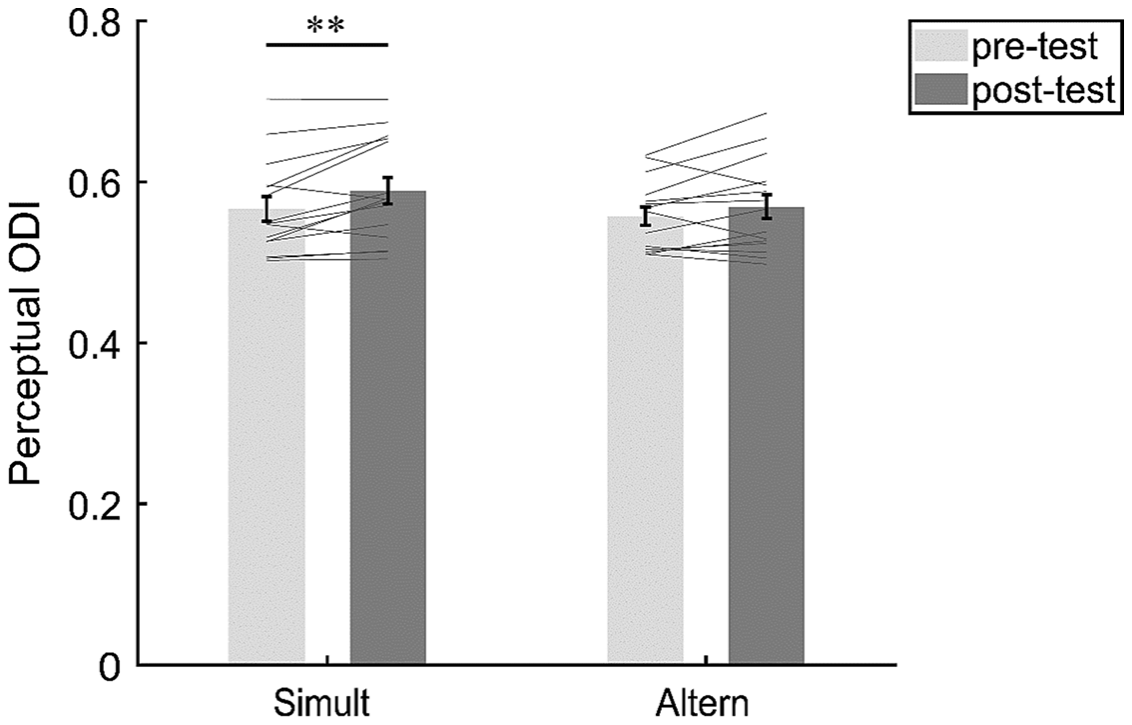
The changes of perceptual ocular dominance in the “Simult” and “Altern” condition. The bars show the grand average ODI between pre- and post-tests in the two conditions. The gray lines show the individual data. Error bars represent standard errors of means. ^**^p < 0.01.

Although the interaction effect was not significant, we were still interested in the changes of ODI under the two stimuli conditions. Therefore, we conducted a post-hoc analysis (Figure 3). The results revealed a significant difference of ODI between the pre-test (*M* = 0.57, *SE* = 0.015) and post-test (*M* = 0.59, *SE* = 0.016; *t*(14) = 3.27, *p* = 0.006, *d* = 0.85, 95% CI = [0.241, 1.427], *BF*_10_ = 9.052, FDR-corrected *p* = 0.011) under the “Simult” condition, suggesting that the perceptual ocular dominance was shifted towards the deprived eye. However, there was no significant difference of ODI between the pre-test (*M* = 0.56, *SE* = 0.011) and post-test (*M* = 0.57, *SE* = 0.015; *t*(14) = 1.61, *p* = 0.130, *d* = 0.42, 95% CI = [−0.119, 0.938], *BF*_10_ = 0.756) under the “Altern” condition.

### 2.3 Discussion

In Experiment 1, we found a shift of ocular dominance towards the deprived eye in the “Simult” condition but not in the “Altern” condition. This seems to show that adaptation of ocular opponency neurons may contribute to the effect of short-term monocular deprivation (at least for phase regularity deprivation).

However, this finding should be interpreted with caution, since we found no significant two-way interaction that strictly examined the difference in the changes of ocular dominance between the two adaptation conditions. In other words, the non-significant interaction result aligned with the homeostatic plasticity mechanism rather than the adaptation of ocular opponency neurons. We speculated that the small sample size might limit the statistical power. Therefore, we used G*Power (Faul et al., 2007) to calculate the minimum required number of participants to detect a significant difference in the deprivation effect between the two conditions. Based on the data collected from 15 participants, we conducted a two-tail paired t-test to compare the monocular deprivation effect (post-test minus pre-test) under the “Simult” condition with that under the “Altern” condition. In G*Power, we calculated an effect size of 0.256, which was then used as a preliminary effect size to calculate required sample size for achieving significance in the monocular deprivation effect between the two conditions. By setting the alpha level at 0.05 and the power at 0.8, we finally calculated that a total of 122 participants would be needed.

In summary, the monocular deprivation effect may result from a combination of the homeostatic plasticity mechanism and ocular opponency neurons adaptation mechanism, with the latter might only having a minor contribution.

## 3 Experiment 2

In Experiment 1, we investigated the role of ocular opponency neuron adaptation in the short-term monocular deprivation effect by reducing interocular competition during the adaptation phase. Unfortunately, no conclusive evidence was found to support or deny the involvement of ocular opponency neurons in the short-term monocular deprivation effect. It should be noted that we did not directly assess the activity of ocular opponency neurons in Experiment 1. Therefore, in order to further investigate the role of ocular opponency neurons in the short-term monocular deprivation effect, we used the SSVEP technique and relied on the intermodulation SSVEP response to directly assess the response of ocular opponency neurons before and after monocular deprivation in Experiment 2.

This approach was based on the pioneering studies that utilized the SSVEP technique to estimate the activities of ocular opponency neurons (Katyal et al., 2016, 2018). Moreover, in our recent work, utilizing the same methodology, we have obtained direct neural evidence supporting the role of ocular opponency neuron adaptation in attention-induced ocular dominance shift (Song et al., 2023a).

To selectively measure the activities of ocular opponency neurons that preferred to a particular eye, we designed three conditions by manipulating the contrast of testing gratings during the binocular rivalry task (Song et al., 2023a). The first was the DE-NDE condition, in which high contrast gratings were presented to the deprived eye and low contrast gratings to the non-deprived eye. The second was the NDE-DE condition, with the non-deprived eye viewing the high contrast gratings and the deprived eye viewing the low contrast gratings. Since high contrast stimuli dominate conscious perception in binocular rivalry (Whittle, 1965; Hollins, 1980), the DE-NDE opponency neurons, which receive excitation from the deprived eye and inhibition from the non-deprived eye, would activate for most of the time in the DE-NDE condition, making their activations the primary source of the intermodulation response in this condition. In comparison, the NDE-DE opponency neurons, which receive excitation from the non-deprived eye and inhibition from the deprived eye, would contribute most to the intermodulation response under the NDE-DE condition. The third condition was the iso-contrast condition, in which equally high contrast gratings were presented to both eyes. This condition was used to measure ocular dominance (Lunghi et al., 2011; Bai et al., 2017; Kim et al., 2017; Lyu et al., 2020; Wang et al., 2020).

Since the responses of the NDE monocular neurons are always stronger than that of the DE monocular neurons during monocular deprivation, we predict that the NDE-DE opponency neurons are always active and subject to sufficient adaptation. As a result, the intermodulation response in the NDE-DE condition is expected to reduce in the post-test as compared with in the pre-test, with a greater reduction than in the DE-NDE condition.

### 3.1 Materials and methods

#### 3.1.1 Participants

Twenty-three participants were recruited for the experiment. In these participants, 20 (10 males, age range 20–28 years) completed the experiment. Among the 3 participants who withdrew midway, two asked to quit in the practicing stage for the binocular rivalry task. One failed in binocular fusion in the EEG pre-test, thus also quit from the rest of the experiment. The sample size was predetermined based on previous studies in this field (Lunghi et al., 2015; Binda et al., 2018; Lyu et al., 2020; Virathone et al., 2021; Baldwin et al., 2022; Kurzawski et al., 2022). All participants were naive to the experimental hypotheses, had normal or corrected-to-normal vision.

#### 3.1.2 Apparatus

The experimental stimuli were programmed in MATLAB and Psychtoolbox (Brainard, 1997; Pelli, 1997). In the practice stage, stimuli were presented on a 27-inch AUS VG279QM LCD monitor with the mean luminance of 30.18 cd/ m^2^, while in the formal test, a 21.5-inch LEN LS2224A LCD monitor with the mean luminance of 31.42 cd/ m^2^ was used. Both monitors were gamma-corrected with the resolution of 1920 × 1,080 pixels and the refresh rate of 60 Hz. Participants viewed the stimuli at a distance of 100 cm in a dimly lit room through a mirror stereoscope. A chinrest was used to stabilize the head.

#### 3.1.3 Stimuli and procedure

##### 3.1.3.1 Binocular rivalry test

The stimuli were two sinusoidal gratings with the Michaelson contrast of 80% or 20% (diameter: 6°, spatial frequency: 0.5 cpd). The orientation of gratings was either 45° clockwise or counterclockwise. The gratings were presented dichoptically at the center of the visual field of each eye. A high contrast checkerboard frame (size: 9° × 9°; thickness: 0.25°) and a central red fixation point (diameter: 0.05°) were presented to both eyes to facilitate binocular fusion. To obtain the SSVEP responses to stimuli from each eye with the frequency-tagging technique, the grating in each eye was phase-reversed flickering (Brown and Norcia, 1997). The flickering frequency was 3 Hz (*f*_1_) for the dominant eye and 3.75 Hz (*f*_2_) for the non-dominant eye (for the definition of eye dominance see Experimental 1).

A binocular rivalry test consisted of fifteen 60-s trials. In each trial, a 5-s blank interval was presented first. Then the rival gratings were presented for 55 s. The orientation and contrast associated to each eye was kept constant within a trial, but randomly varied across the trials. The contrast of the gratings could be as follows: (1) 80% contrast in the dominant eye, 20% contrast in the non-dominant eye; (2) 20% contrast in the dominant eye, 80% contrast in the non-dominant eye; (3) 80% contrast in both eyes. Participants were instructed to hold down one of the three keys (Right, Left, or Down Arrow) to report their perceptions (clockwise, counterclockwise, or mixed).

##### 3.1.3.2 Monocular deprivation

Participants wore an eye-patch made of parchment paper over the dominant eye or the non-dominant eye (counter-balanced across the participants) for 2.5 h. All the edges of this translucent eye-patch were adhered to the skin, allowing the light to reach the eye but completely preventing pattern vision. During the deprivation, participants were free to perform normal activities (e.g., using the mobile phone).

#### 3.1.4 Experimental design

Before the formal experiment, all participants practiced the binocular rivalry task similarly to Experiment 1. For consistency between the practice and the formal tests in Experiment 2, the gratings used in the practice were also phase-reversed flickering.

**Figure 4:**
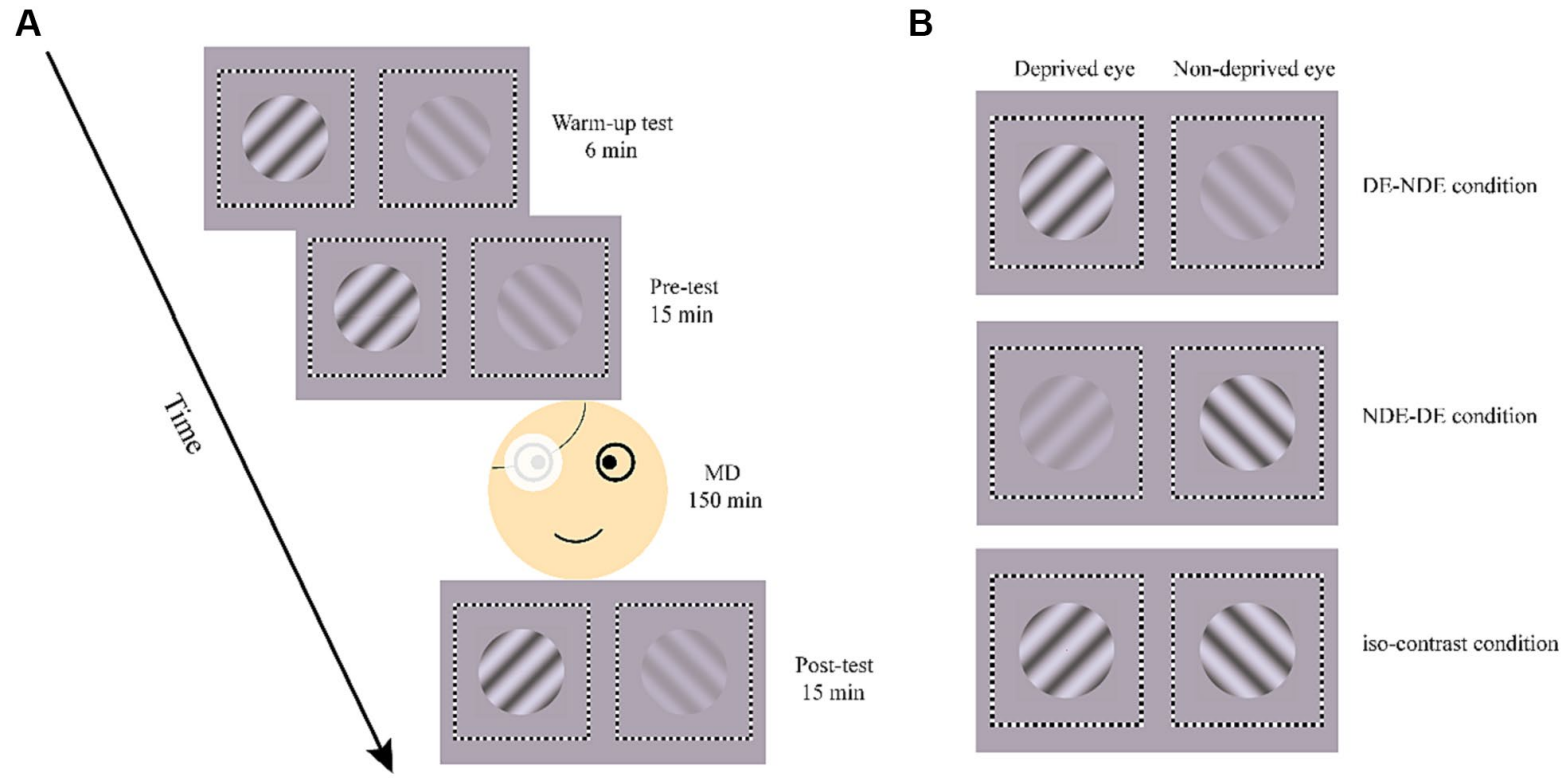
Illustration of the process in formal experiment **(A)** and the three test conditions **(B)**. MD, monocular deprivation.

In the formal experiment, participants first performed a warm-up test consisting of six trials, the data of which were not analyzed (Bai et al., 2017; Bao et al., 2018). Then, a binocular rivalry pre-test including 15 trials was performed, which measured both the perceptual ocular dominance and SSVEP responses before monocular deprivation. This was followed by 2.5 h of monocular deprivation. Immediately after the monocular deprivation, a 15-trial binocular rivalry test was performed as the post-test (Figure 4A).

Each binocular rivalry test consisted of three conditions, determined by the contrast of the grating in each eye (Figure 4B). In the DE-NDE condition, the higher contrast (80%) gratings were presented to the deprived eye, and the lower contrast (20%) gratings to the non-deprived eye. In the NDE-DE condition, the non-deprived eye viewed the higher contrast gratings, while the deprived eye viewed the lower contrast gratings. There was also an iso-contrast condition, where 80% contrast gratings were presented to both eyes. In the pre- and post-test, each condition consisted of 5 trials. The order of conditions was counterbalanced across trials but remained consistent between the pre- and post-test.

#### 3.1.5 EEG data acquisition

The continuous EEG was recorded using the Neuroscan Synamps2 system with a 64-channel Ag/AgCl electrode cap mounted according to the International 10–20 system. All electrodes were referenced to the left mastoid (M1). Vertical and horizontal electrooculograms (VEOG and HEOG) were recorded to monitor blinks and eye movements. The EEG recording was digitized at 1000 Hz with a band-pass filter (0.05–100 Hz), including a 50 Hz notch filter. Impedances were kept below 5 kΩ.

#### 3.1.6 Data analysis

##### 3.1.6.1 EEG preprocessing

Offline analysis was performed using customized MATLAB codes and FieldTrip (Oostenveld et al., 2011). The electrodes for M1, M2 and EOGs were initially extracted from the raw EEG data. The data were then segmented according to the trial periods and resampled to 1,024 Hz before being band-pass filtered between 0.5 and 30 Hz. Next, the surface Laplacian spatial filter was employed to effectively reduce common noise (Hjorth, 1975), by subtracting the average signal of the nearest four to eight electrodes from the central electrode’s signal.

##### 3.1.6.2 Extraction of SSVEP signals

**Figure 5:**
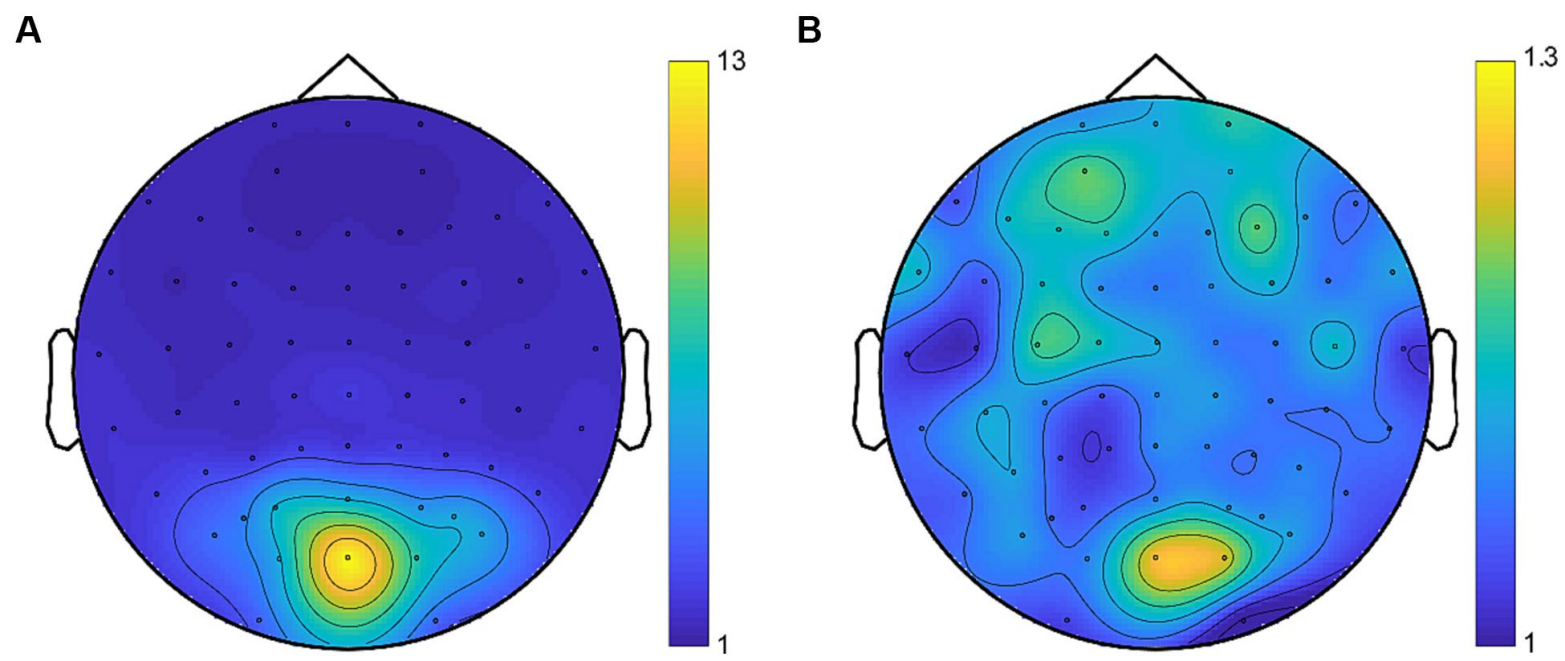
Average topographies for SNR at the fundamental frequencies **(A)** and the intermodulation frequency **(B)**. Despite the lower SNR of intermodulation responses compared to the fundamental responses, the topographies consistently revealed activity in the occipital region.

Fast Fourier transform (FFT) was applied to the preprocessed time series. We extracted the SSVEP signals of the even harmonics of the phase-reversed flickering frequency in the response spectrum (6 Hz (2*f*_1_) and 7.5 Hz (2*f*_2_)) as the SSVEP responses at the fundamental frequencies (Norcia et al., 2015; Dong et al., 2020) which tagged the monocular neural activities for each eye. Moreover, we extracted the SSVEP signals at the intermodulation frequencies which were considered to reflect the neural activities of interocular competition (Poggio and Talbot, 1981; May et al., 2012; Said and Heeger, 2013; Katyal et al., 2016, 2018; Du et al., 2023). Consistent with previous research (Govenlock et al., 2008; Cai et al., 2020; Gu et al., 2020; Mersad and Caristan, 2021; Song et al., 2023a; Yan et al., 2023), intermodulation responses were identified at the commonly used frequency of 6.75 Hz (f1 + f2), exhibiting a superior response signal. The signal-to-noise ratio (SNR) was calculated by dividing the power at the extracted frequency by the mean power of the 20 surrounding (10 on each side, excluding the immediately adjacent bin) frequency bins (Zhang et al., 2011; Lyu et al., 2020; Song et al., 2023b). The scalp topographies of SNR are depicted in Figure 5. The SSVEP amplitude at the frequencies of interest (i.e., 2*f*_1_, 2*f*_2_, and *f*_1_ + *f*_2_) was computed using an adaptive recursive least square (RLS) filter (Tang and Norcia, 1995) with a 1-s sliding window (Zhang et al., 2011). The initial 2 s of data were excluded from the analysis to avoid the start-up transient of the adaptive filter (Tang and Norcia, 1995; Zhang et al., 2011). The remaining time course was averaged to estimate the SSVEP amplitude in the given trial. According to prior researches (Zhang et al., 2011; Gu et al., 2020; Song et al., 2023a), the SSVEP amplitudes were averaged across electrode OZ and its five neighboring electrodes (POZ, O1, O2, CB1 and CB2) for statistical comparisons.

##### 3.1.6.3 Index defining the ocular dominance

To quantify perceptual and neural eye dominance, we calculated the ODI on both the dynamics of binocular rivalry and the SSVEP amplitudes of the fundamental frequencies in the iso-contrast condition for the pre- and post-test. The ODI was calculated similarly to Experiment 1 using the following formula. In all cases, the same formula was applied to psychophysical and EEG data.


$$ODI=\frac{y_{DE}}{y_{NDE}+y_{DE}}$$

For psychophysics, *y* represented the summed phase durations for exclusive perception of the stimulus in the deprived or non-deprived eye as in Experiment 1; for EEG, *y* represented the mean SSVEP amplitudes to stimuli in the deprived or non-deprived eye.

##### 3.1.6.4 Statistical analyses

Statistical analyses were performed using MATLAB. To investigate the effects of short-term monocular deprivation on ocular dominance, the paired-sample *t*-tests was performed to compare the ODI between the pre-test and the post-test. Moreover, the intermodulation responses of the DE-NDE condition and the NDE-DE condition were compared across pre- and post-tests using a 2 (test phase: pre-test, post-test) × 2 (condition: DE-NDE condition, NDE-DE condition) repeated measures ANOVA. All t-tests were two-tailed and α value of 0.05 was used. As outlined in Experiment 1, Bayesian analysis was similarly performed using JASP with default priors. The Bayesian paired-sample *t*-tests were conducted to calculate the *BF*_10_, while the Bayesian repeated measures ANOVA involved the computation of *BF*_incl_.

### 3.2 Results

#### 3.2.1 Behavioral results

**Figure 6:**
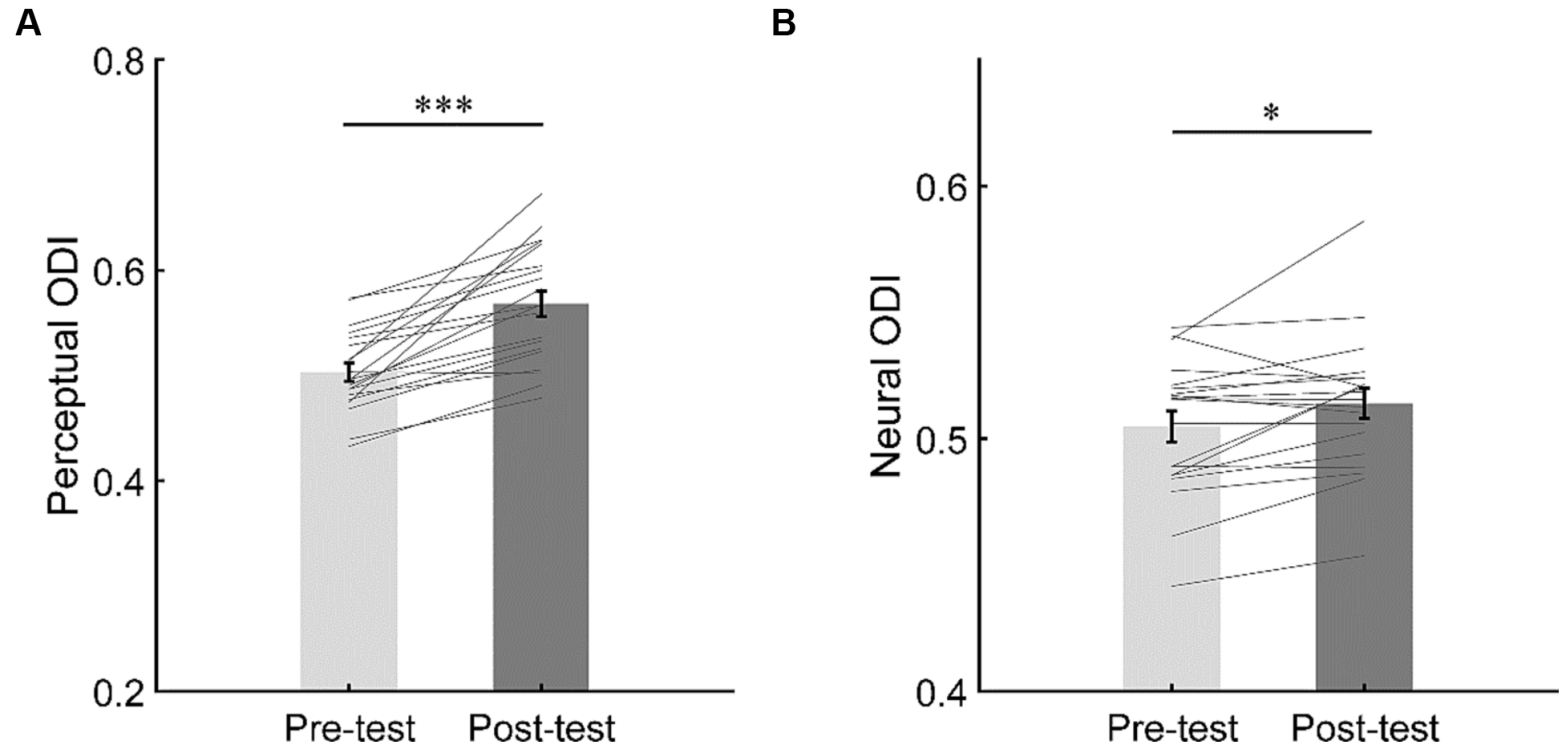
The changes of perceptual ocular dominance **(A)** and neural ocular dominance **(B)**. The bars show the grand average ODI in the iso-contrast condition for pre- and post-tests. The gray lines show the individual data. Error bars represent standard errors of means. ^***^p < 0.001; ^*^p < 0.05.

The perceptual ODI was greater in the post-test (*M* = 0.57, *SE* = 0.012) than in the pre-test (*M* = 0.50, *SE* = 0.009; *t*(19) = 6.45, *p* < 0.001, *d* = 1.44, 95% CI = [0.04, 0.09], *BF*_10_ > 100), suggesting that short-term monocular deprivation shifted the perceptual ocular dominance towards the deprived eye (Figure 6A).

#### 3.2.2 EEG results

To evaluate the shift of neural ocular dominance, a paired *t*-test was performed on the neural ODI between the pre- and post-tests. The results showed that the neural ODI was greater in the post-test (*M* = 0.51, *SE* = 0.006) than in the pre-test (*M* = 0.50, *SE* = 0.006; *t*(19) = 2.58, *p* = 0.018, *d* = 0.58, 95% CI = [0.002, 0.017], *BF*_10_ = 3.10), suggesting that short-term monocular deprivation also shifted the neural ocular dominance towards the deprived eye (Figure 6B).

**Figure 7:**
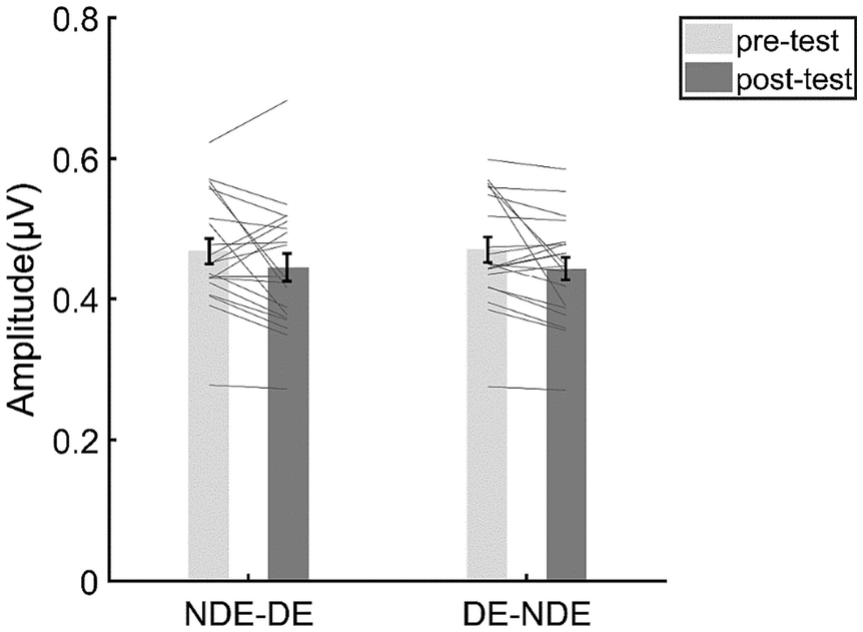
The results for the SSVEP amplitudes at the intermodulation frequencies. The bars show the grand average SSVEP amplitudes for each condition. The gray lines show the individual data. Error bars represent standard errors of means.

To examine the response change of opponency neurons, we then focused on the SSVEP responses at the intermodulation frequencies in the DE-NDE condition and the NDE-DE condition. The results of the repeated measurements ANOVA (Figure 7) showed that the main effect of test phase was not significant (*F*(1,19) = 3.99, *p* = 0.060, η^2^ = 0.17, *BF*_incl_ = 1.42). Moreover, the main effect of condition was not significant (*F*(1,19) < 0.001, *p* = 0.98, *η*^2^ < 0.001, *BF*_incl_ = 0.35). More importantly, the interaction between the test phase and the condition was not significant (*F*(1,19) = 0.32, *p* = 0.58, *η*^2^ = 0.02), either. Consistent with this, the Bayesian ANOVA yields moderate evidence (*BF*_incl_ = 0.31, that is to say a BF between 1/10 and 1/3) supporting the null hypothesis of no interaction effect between the test phase and the stimulus condition.

### 3.3 Discussion

In Experiment 2, we further explored the contribution of the adaptation of ocular opponency neurons to the short-term monocular deprivation effect by directly assessing the response of ocular opponency neurons using the SSVEP technique. Consistent with previous findings, we found a shift of perceptual and neural ocular dominance to the deprived eye after 2.5 h of monocular deprivation. However, the SSVEP response at the intermodulation frequencies (i.e., intermodulation response) did not exhibit significant alteration following the monocular deprivation. Therefore, the present experiment did not find sufficient evidence to support the involvement of ocular opponency neurons in the short-term monocular deprivation effect.

A plausible account for the negative results regarding to intermodulation response could be the presence of noise during data collection and analysis procedures. However, it should be emphasized that the data collection environment, equipment, and data analysis methods employed in this experiment were consistent with those utilized in our recent research (Song et al., 2023a), which provided direct neural evidence supporting the role of ocular opponency neuron adaptation in the attention-induced ocular dominance shift. As measured by intermodulation responses in that study, the opponency neurons preferring to the attended eye showed a greater decrease of activity compared to those preferring to the unattended eye following the adaptation with attention biased towards the attended eye. This finding suggested that the ocular opponency neurons receiving excitatory inputs from the attended eye underwent a greater degree of adaptation, causing their reduced activity during the post-test. According to the ocular-opponency-neuron model (Said and Heeger, 2013), this would lead to a decrease in the inhibition received by the unattended eye, resulting in a ocular dominance shift towards the unattended eye. Therefore, the finding in Song et al. (2023a) can serve as a positive control for the current experiment, suggesting that the negative results obtained were not due to extraneous noise introduced during data collection or analysis.

## 4 General discussion

In the current study, we investigated the role of the adaptation of ocular opponency neurons in the short-term monocular deprivation effect from two perspectives. On one hand, we developed two variants of the “pink-noise” monocular deprivation paradigm, allowing the adaptation of ocular opponency neurons either balanced or imbalanced across the two eyes. On the other hand, we employed the conventional monocular patching deprivation and directly evaluated the response of ocular opponency neurons before and after deprivation using the SSVEP techniques. However, consistent with each other, both experiments failed to provide strong evidence supporting the involvement of ocular opponency neuron adaptation in the short-term monocular deprivation effect. Therefore, the current study suggests that the adaptation of ocular opponency neurons may not play a predominant role in the short-term monocular deprivation effect, though the existence of a minor (yet negligible) effect is still possible.

It should be noted that it is not our first time to pay attention to the potential role of ocular opponency neuron adaptation in short-term ocular dominance plasticity. Other than monocular deprivation, our work with dichoptic-backward-movie adaptation has proposed that adaptation of ocular opponency neurons may mediate the shift of ocular dominance led by prolonged eye-based attention bias (Song et al., 2023b), which is later supported by SSVEP evidence (Song et al., 2023a). However, unlike these studies, the present research did not provide evidence supporting the involvement of the ocular opponency neuron adaptation in short-term monocular deprivation effects. Therefore, it is possible that the adaptation of ocular opponency neurons may only modulate attention-induced ocular dominance shift.

Why does the ocular opponency neuron adaptation selectively modulate attention-induced ocular dominance plasticity but nearly not impact the short-term monocular deprivation effect? One possible explanation is that the role of homeostatic plasticity mechanisms may depend on imbalanced monocular responses of the eyes. Previous studies have revealed a profound discrepancy in monocular visual responses between the two eyes during monocular deprivation (Lyu et al., 2020), but only a small difference during the dichoptic-backward-movie adaptation (Song et al., 2023b). According to the homeostatic plasticity mechanism, the homeostatic regulating process is only triggered when there exists a significant discrepancy in neural responses between the two eyes (Keck et al., 2017). Therefore, the homeostatic plasticity mechanism is believed to deeply involved in monocular deprivation, but not necessarily work or take an important effect during the dichoptic-backward-movie adaptation. Consequently, the role of ocular opponency neurons may be clouded by that of the homeostatic plasticity mechanism during monocular deprivation. However, this is no longer a problem during the dichoptic-backward-movie adaptation, where both eyes received comparable sensory inputs.

While the concept of ocular opponency neuron has well been established and the theoretical model elucidating their roles in binocular rivalry has been devised in 2013 (Poggio and Talbot, 1981; May et al., 2012; Said and Heeger, 2013), to our knowledge, the direct electrophysiological evidence supporting the existence of ocular opponency neurons is still lacking. However, considering that ocular opponency neurons are responsible for interocular-difference calculations, we may speculate the neural locus of ocular opponency neurons along the visual pathway based on this key function. The geniculate-cortical visual pathway is a highly directional system with well-specified connections (Julesz, 1971). The visual information flows of both eyes remain independent of each other only during very early processing stages. After visual information passes from the retina through the optic chiasm, monocular selective neurons only exist in the lateral geniculate nucleus (LGN) and primary visual cortex (V1). The first station the visual information flow reaches the cortex is layer 4 in V1, where it forms functional columns that are selective for each eye. During the transmission from the V1 middle layer to the surface layer, binocular information starts to be extracted by various interneurons to integrate both monocular information and establish the stereopsis (Poggio, 1984; Julesz, 1986). Next, from the V1 surface layer to the extrastriate cortex, neurons representing the eye-of-origin are thought to gradually disappear (Le Vay et al., 1980; Crowley and Katz, 2002). Therefore, we presume, ocular opponency neurons might be located within the V1 middle layer to the surface layer. Would any later stage be a possible locus? Previous monkey studies have shown that the eye dominance in binocular rivalry could also be represented by neurons in relatively later stages including V4 and MT, the middle temporal visual area (Logothetis and Schall, 1989a,b; Leopold and Logothetis, 1996). A conventional explanation is that the monocular information may be partly preserved to higher levels of the visual pathway (Ramachandran, 1991), for example, via the direct projection from the V1 layer 4B and 6 to MT (Shipp and Zeki, 1989); but an alternative account considers the perceptual alternation not only as the eye-dominance rivalry, but more as the pattern rivalry of stimuli arising from the excitation and inhibition of the neuron clusters in higher visual areas representing these patterns (Logothetis, 1998), which hence has much less to do with the interocular-difference calculations discussed here. Therefore, we are more inclined to favor the early locus of ocular opponency neurons, which is still to be examined by future single-unit work.

To conclude, our results suggest that ocular opponency neurons may not play a significant role in the formation of short-term monocular deprivation effect. Instead, we believe that the homeostatic plasticity mechanism is still predominantly responsible for this type of ocular dominance plasticity (Lunghi et al., 2015; Bai et al., 2017; Binda et al., 2018; Min et al., 2019; Zhou et al., 2019; Lyu et al., 2020).

## Data availability statement

The raw data supporting the conclusions of this article will be made available by the authors, without undue reservation.

## Ethics statement

The studies involving humans were approved by Institutional Review Board of the Institute of Psychology, Chinese Academy of Sciences. The studies were conducted in accordance with the local legislation and institutional requirements. The participants provided their written informed consent to participate in this study.

## Author contributions

JW: Formal analysis, Investigation, Methodology, Software, Writing – original draft. FS: Formal analysis, Investigation, Methodology, Software, Writing – original draft. XH: Investigation, Writing – review & editing. MB: Conceptualization, Funding acquisition, Supervision, Writing – review & editing.

## References

[ref1] Bai J. Dong X. He S. Bao M. (2017). Monocular deprivation of Fourier phase information boosts the deprived eye's dominance during interocular competition but not interocular phase combination. Neuroscience 352, 122–130. doi: 10.1016/j.neuroscience.2017.03.053, PMID: 28391010

[ref2] Baldwin A. S. Finn A. E. Green H. M. Gant N. Hess R. F. (2022). Exercise does not enhance short-term deprivation-induced ocular dominance plasticity: evidence from dichoptic surround suppression. Vis. Res. 201:108123. doi: 10.1016/j.visres.2022.108123 36193605

[ref3] Bang J. W. Hamilton-Fletcher G. Chan K. C. (2023). Visual plasticity in adulthood: perspectives from Hebbian and homeostatic plasticity. Neuroscientist 29, 117–138. doi: 10.1177/10738584211037619, PMID: 34382456 PMC9356772

[ref4] Bao M. Dong B. Liu L. Engel S. A. Jiang Y. (2018). The best of both worlds: adaptation during natural tasks produces long-lasting plasticity in perceptual ocular dominance. Psychol. Sci. 29, 14–33. doi: 10.1177/0956797617728126 29160741

[ref5] Baroncelli L. Lunghi C. (2021). Neuroplasticity of the visual cortex: in sickness and in health. Exp. Neurol. 335:113515. doi: 10.1016/j.expneurol.2020.113515 33132181

[ref6] Binda P. Kurzawski J. W. Lunghi C. Biagi L. Tosetti M. Morrone M. C. (2018). Response to short-term deprivation of the human adult visual cortex measured with 7T BOLD. elife 7:e40014. doi: 10.7554/eLife.40014 30475210 PMC6298775

[ref7] Brainard D. H. (1997). The psychophysics toolbox. Spat. Vis. 10, 433–436. doi: 10.1163/156856897x00357 9176952

[ref8] Brown R. J. Norcia A. M. (1997). A method for investigating binocular rivalry in real-time with the steady-state VEP. Vis. Res. 37, 2401–2408. doi: 10.1016/s0042-6989(97)00045-x, PMID: 9381675

[ref9] Cai Y. Mao Y. Ku Y. Chen J. (2020). Holistic integration in the processing of Chinese characters as revealed by electroencephalography frequency tagging. Perception 49, 658–671. doi: 10.1177/0301006620929197, PMID: 32552487

[ref10] Crowley J. C. Katz L. C. (2002). Ocular dominance development revisited. Curr. Opin. Neurobiol. 12, 104–109. doi: 10.1016/s0959-4388(02)00297-0, PMID: 11861172

[ref11] Dong X. Du X. Bao M. (2020). Repeated contrast adaptation does not cause habituation of the adapter. Front. Hum. Neurosci. 14:589634. doi: 10.3389/fnhum.2020.589634, PMID: 33424564 PMC7785701

[ref12] Du X. Liu L. Dong X. Bao M. (2023). Effects of altered-reality training on interocular disinhibition in amblyopia. Ann. N. Y. Acad. Sci. 1522, 126–138. doi: 10.1111/nyas.14969 36811156

[ref13] Faul F. Erdfelder E. Lang A. G. Buchner A. (2007). G*power 3: a flexible statistical power analysis program for the social, behavioral, and biomedical sciences. Behav. Res. Methods 39, 175–191. doi: 10.3758/bf03193146 17695343

[ref14] Govenlock S. Kliegl K. Sekuler A. Bennett P. (2008). Assessing the effect of aging on orientation selectivity of visual mechanisms with the steady state visually evoked potential. J. Vis. 8:424. doi: 10.1167/8.6.424

[ref15] Gu L. Deng S. Feng L. Yuan J. Chen Z. Yan J.. (2020). Effects of monocular perceptual learning on binocular visual processing in adolescent and adult amblyopia. iScience 23:100875. doi: 10.1016/j.isci.2020.100875, PMID: 32062454 PMC7021554

[ref16] Hjorth B. (1975). An on-line transformation of EEG scalp potentials into orthogonal source derivations. Electroencephalogr. Clin. Neurophysiol. 39, 526–530. doi: 10.1016/0013-4694(75)90056-5 52448

[ref17] Hollins M. (1980). The effect of contrast on the completeness of binocular rivalry suppression. Percept. Psychophys. 27, 550–556. doi: 10.3758/bf03198684, PMID: 7393703

[ref18] Huang X. Xia H. Zhang Q. Blakemore C. Nan Y. Wang W.. (2022). New treatment for amblyopia based on rules of synaptic plasticity: a randomized clinical trial. Sci. China Life Sci. 65, 451–465. doi: 10.1007/s11427-021-2030-6 35015247

[ref19] Julesz B. (1971). Foundations of Cyclopean Perception. Chicago, IL: University of Chicago Press.

[ref20] Julesz B. (1986). Stereoscopic vision. Vis. Res. 26, 1601–1612. doi: 10.1016/0042-6989(86)90178-1 3303677

[ref21] Katyal S. Engel S. A. He B. He S. (2016). Neurons that detect interocular conflict during binocular rivalry revealed with EEG. J. Vis. 16:18. doi: 10.1167/16.3.18, PMID: 26891825

[ref22] Katyal S. Vergeer M. He S. He B. Engel S. A. (2018). Conflict-sensitive neurons gate interocular suppression in human visual cortex. Sci. Rep. 8:1239. doi: 10.1038/s41598-018-19809-w, PMID: 29352155 PMC5775389

[ref23] Keck T. Toyoizumi T. Chen L. Doiron B. Feldman D. E. Fox K.. (2017). Integrating Hebbian and homeostatic plasticity: the current state of the field and future research directions. Philos. Trans. R. Soc. Lond. Ser. B Biol. Sci. 372:20160158. doi: 10.1098/rstb.2016.0158, PMID: 28093552 PMC5247590

[ref24] Kim H. W. Kim C. Y. Blake R. (2017). Monocular perceptual deprivation from interocular suppression temporarily imbalances ocular dominance. Curr. Biol. 27, 884–889. doi: 10.1016/j.cub.2017.01.063, PMID: 28262490

[ref25] Kurzawski J. W. Lunghi C. Biagi L. Tosetti M. Morrone M. C. Binda P. (2022). Short-term plasticity in the human visual thalamus. elife 11:e74565. doi: 10.7554/eLife.74565 35384840 PMC9020816

[ref26] Le Vay S. Wiesel T. N. Hubel D. H. (1980). The development of ocular dominance columns in normal and visually deprived monkeys. J. Comp. Neurol. 191, 1–51. doi: 10.1002/cne.901910102, PMID: 6772696

[ref27] Leopold D. A. Logothetis N. K. (1996). Activity changes in early visual cortex reflect monkeys' percepts during binocular rivalry. Nature 379, 549–553. doi: 10.1038/379549a0, PMID: 8596635

[ref28] Logothetis N. K. (1998). Single units and conscious vision. Philos. Trans. R. Soc. Lond. B Biol. Sci. 353, 1801–1818. doi: 10.1098/rstb.1998.0333 9854253 PMC1692419

[ref29] Logothetis N. K. Schall J. D. (1989a). “Motion perception related activity in the middle temporal visual area (MT) of the macaque monkey” in Neural Mechanisms of Visual Perception: Proceedings of the Second Retina Research Foundation Symposium. eds. Lam D. M.-K. Gilbert C. D. (Woodlands, TX: Portfolio Publishing), 199–222.

[ref30] Logothetis N. K. Schall J. D. (1989b). Neuronal correlates of subjective visual perception. Science (New York, N.Y.) 245, 761–763. doi: 10.1126/science.2772635 2772635

[ref31] Lunghi C. Berchicci M. Morrone M. C. Di Russo F. (2015). Short-term monocular deprivation alters early components of visual evoked potentials. J. Physiol. 593, 4361–4372. doi: 10.1113/JP270950, PMID: 26119530 PMC4594246

[ref32] Lunghi C. Burr D. C. Morrone C. (2011). Brief periods of monocular deprivation disrupt ocular balance in human adult visual cortex. Curr. Biol. 21, R538–R539. doi: 10.1016/j.cub.2011.06.004, PMID: 21783029

[ref33] Lunghi C. Burr D. C. Morrone M. C. (2013). Long-term effects of monocular deprivation revealed with binocular rivalry gratings modulated in luminance and in color. J. Vis. 13:1. doi: 10.1167/13.6.1 23637272

[ref34] Lyu L. He S. Jiang Y. Engel S. A. Bao M. (2020). Natural-scene-based steady-state visual evoked potentials reveal effects of short-term monocular deprivation. Neuroscience 435, 10–21. doi: 10.1016/j.neuroscience.2020.03.039 32229234

[ref35] Maffei A. Nelson S. B. Turrigiano G. G. (2004). Selective reconfiguration of layer 4 visual cortical circuitry by visual deprivation. Nat. Neurosci. 7, 1353–1359. doi: 10.1038/nn1351, PMID: 15543139

[ref36] May K. A. Zhaoping L. Hibbard P. B. (2012). Perceived direction of motion determined by adaptation to static binocular images. Curr. Biol. 22, 28–32. doi: 10.1016/j.cub.2011.11.025, PMID: 22177901

[ref37] Mersad K. Caristan C. (2021). Blending into the crowd: electrophysiological evidence of gestalt perception of a human dyad. Neuropsychologia 160:107967. doi: 10.1016/j.neuropsychologia.2021.107967 34303717

[ref38] Min S. H. Baldwin A. S. Hess R. F. (2019). Ocular dominance plasticity: a binocular combination task finds no cumulative effect with repeated patching. Vis. Res. 161, 36–42. doi: 10.1016/j.visres.2019.05.007, PMID: 31194984

[ref39] Min S. H. Baldwin A. S. Reynaud A. Hess R. F. (2018). The shift in ocular dominance from short-term monocular deprivation exhibits no dependence on duration of deprivation. Sci. Rep. 8:17083. doi: 10.1038/s41598-018-35084-1, PMID: 30459412 PMC6244356

[ref40] Min S. H. Mao Y. Chen S. Hess R. F. Zhou J. (2022). Modulation of mean luminance improves binocular balance across spatial frequencies in amblyopia. iScience 25:104598. doi: 10.1016/j.isci.2022.104598, PMID: 35789838 PMC9249912

[ref41] Nguyen B. N. Malavita M. Carter O. L. McKendrick A. M. (2021). Neuroplasticity in older adults revealed by temporary occlusion of one eye. Cortex 143, 1–11. doi: 10.1016/j.cortex.2021.07.004, PMID: 34365199

[ref42] Norcia A. M. Appelbaum L. G. Ales J. M. Cottereau B. R. Rossion B. (2015). The steady-state visual evoked potential in vision research: a review. J. Vis. 15:4. doi: 10.1167/15.6.4, PMID: 26024451 PMC4581566

[ref43] Oostenveld R. Fries P. Maris E. Schoffelen J. M. (2011). FieldTrip: open source software for advanced analysis of MEG, EEG, and invasive electrophysiological data. Comput. Intell. Neurosci. 2011:156869. doi: 10.1155/2011/156869, PMID: 21253357 PMC3021840

[ref44] Pelli D. G. (1997). The VideoToolbox software for visual psychophysics: transforming numbers into movies. Spat. Vis. 10, 437–442. doi: 10.1163/156856897x00366 9176953

[ref45] Poggio G. F. (1984). “Processing of stereoscopic information in primate visual cortex” in Dynamical Aspects of Neocortical Function. eds. Edelman G. M. Gall W. E. Cowan W. M. (New York, NY: Wiley), 613–635.

[ref46] Poggio G. F. Talbot W. H. (1981). Mechanisms of static and dynamic stereopsis in foveal cortex of the rhesus monkey. J. Physiol. 315, 469–492. doi: 10.1113/jphysiol.1981.sp013759, PMID: 7310720 PMC1249394

[ref47] Ramachandran V. S. (1991). Form, motion, and binocular rivalry. Science 251, 950–951. doi: 10.1126/science.2000497 2000497

[ref48] Ramamurthy M. Blaser E. (2018). Assessing the kaleidoscope of monocular deprivation effects. J. Vis. 18:14. doi: 10.1167/18.13.14, PMID: 30572342

[ref49] Said C. P. Heeger D. J. (2013). A model of binocular rivalry and cross-orientation suppression. PLoS Comput. Biol. 9:e1002991. doi: 10.1371/journal.pcbi.1002991 23555225 PMC3610603

[ref50] Sheynin Y. Chamoun M. Baldwin A. S. Rosa-Neto P. Hess R. F. Vaucher E. (2019). Cholinergic potentiation alters perceptual eye dominance plasticity induced by a few hours of monocular patching in adults. Front. Neurosci. 13:22. doi: 10.3389/fnins.2019.00022, PMID: 30766471 PMC6365463

[ref51] Shipp S. Zeki S. (1989). The organization of connections between areas V5 and V1 in macaque monkey visual cortex. Eur. J. Neurosci. 1, 309–332. doi: 10.1111/j.1460-9568.1989.tb00798.x 12106142

[ref52] Song F. Lyu L. Bao M. (2023a). Adaptation of ocular opponency neurons mediates attention-induced ocular dominance plasticity. Neurosci. Bull. doi: 10.1007/s12264-023-01103-z [Epub ahead of print]. PMC10912405 37635196

[ref53] Song F. Lyu L. Zhao J. Bao M. (2023b). The role of eye-specific attention in ocular dominance plasticity. Cereb. Cortex 33, 983–996. doi: 10.1093/cercor/bhac116, PMID: 35332915 PMC9930618

[ref54] Spiegel D. P. Byblow W. D. Hess R. F. Thompson B. (2013). Anodal transcranial direct current stimulation transiently improves contrast sensitivity and normalizes visual cortex activation in individuals with amblyopia. Neurorehabil. Neural Repair 27, 760–769. doi: 10.1177/1545968313491006, PMID: 23774122

[ref55] Tang Y. Norcia A. M. (1995). An adaptive filter for steady-state evoked responses. Electroencephalogr. Clin. Neurophysiol. 96, 268–277. doi: 10.1016/0168-5597(94)00309-3, PMID: 7750452

[ref56] Tuna A. R. Pinto N. Fernandes A. Brardo F. M. Pato M. V. (2022). Theta burst stimulation in adults with symmetric and asymmetric visual acuity. Int. Ophthalmol. 42, 2785–2799. doi: 10.1007/s10792-022-02269-7, PMID: 35353292

[ref57] Turrigiano G. G. (1999). Homeostatic plasticity in neuronal networks: the more things change, the more they stay the same. Trends Neurosci. 22, 221–227. doi: 10.1016/s0166-2236(98)01341-1, PMID: 10322495

[ref58] Turrigiano G. (2011). Too many cooks? Intrinsic and synaptic homeostatic mechanisms in cortical circuit refinement. Annu. Rev. Neurosci. 34, 89–103. doi: 10.1146/annurev-neuro-060909-153238, PMID: 21438687

[ref59] Turrigiano G. G. Nelson S. B. (2004). Homeostatic plasticity in the developing nervous system. Nat. Rev. Neurosci. 5, 97–107. doi: 10.1038/nrn1327 14735113

[ref60] van den Bergh D. Wagenmakers E.-J. Aust F. (2023). Bayesian repeated-measures analysis of variance: an updated methodology implemented in JASP. Adv. Methods Pract. Psychol. Sci. 6:25152459231168024. doi: 10.1177/25152459231168024

[ref61] van Doorn J. van den Bergh D. Böhm U. Dablander F. Derks K. Draws T.. (2021). The JASP guidelines for conducting and reporting a Bayesian analysis. Psychon. Bull. Rev. 28, 813–826. doi: 10.3758/s13423-020-01798-5, PMID: 33037582 PMC8219590

[ref62] Virathone L. Nguyen B. N. Dobson F. Carter O. L. McKendrick A. M. (2021). Exercise alone impacts short-term adult visual neuroplasticity in a monocular deprivation paradigm. J. Vis. 21:12. doi: 10.1167/jov.21.11.12, PMID: 34668930 PMC8543434

[ref63] Wagenmakers E. J. Love J. Marsman M. Jamil T. Ly A. Verhagen J.. (2018). Bayesian inference for psychology. Part II: example applications with JASP. Psychon. Bull. Rev. 25, 58–76. doi: 10.3758/s13423-017-1323-7, PMID: 28685272 PMC5862926

[ref64] Wang M. McGraw P. Ledgeway T. (2020). Short-term monocular deprivation reduces inter-ocular suppression of the deprived eye. Vis. Res. 173, 29–40. doi: 10.1016/j.visres.2020.05.001 32460171

[ref65] Wang M. McGraw P. Ledgeway T. (2021). Attentional eye selection modulates sensory eye dominance. Vis. Res. 188, 10–25. doi: 10.1016/j.visres.2021.06.006, PMID: 34280813

[ref66] Whittle P. (1965). Binocular rivalry and the contrast at contours. Q. J. Exp. Psychol. 17, 217–226. doi: 10.1080/17470216508416435 5825693

[ref67] Wiesel T. N. Hubel D. H. (1963). Single-cell responses in striate cortex of kittens deprived of vision in one eye. J. Neurophysiol. 26, 1003–1017. doi: 10.1152/jn.1963.26.6.1003 14084161

[ref68] Yan X. Chen J. Fu Y. Wu Y. Ku Y. Cao F. (2023). Orthographic deficits but typical visual perceptual processing in Chinese adults with reading disability. bioRxiv [Preprint]. doi: 10.1101/2023.02.13.528424

[ref69] Yao Z. He Z. Wang Y. Lu F. Qu J. Zhou J.. (2017). Absolute not relative interocular luminance modulates sensory eye dominance plasticity in adults. Neuroscience 367, 127–133. doi: 10.1016/j.neuroscience.2017.10.029, PMID: 29111363

[ref70] Zhang P. Jamison K. Engel S. He B. He S. (2011). Binocular rivalry requires visual attention. Neuron 71, 362–369. doi: 10.1016/j.neuron.2011.05.035, PMID: 21791293 PMC3175243

[ref71] Zhou J. Clavagnier S. Hess R. F. (2013). Short-term monocular deprivation strengthens the patched eye's contribution to binocular combination. J. Vis. 13:12. doi: 10.1167/13.5.12, PMID: 23599416

[ref72] Zhou J. He Z. Wu Y. Chen Y. Chen X. Liang Y.. (2019). Inverse occlusion: a binocularly motivated treatment for amblyopia. Neural Plast. 2019, 1–12. doi: 10.1155/2019/5157628 PMC6444262 31015829

